# International expert consensus statement about methods and indications for keyhole microneurosurgery from International Society on Minimally Invasive Neurosurgery

**DOI:** 10.1007/s10143-019-01188-z

**Published:** 2019-11-21

**Authors:** Qing Lan, Michael Sughrue, Nikolai J. Hopf, Kentaro Mori, Jaechan Park, Hugo Andrade-Barazarte, Mangaleswaran Balamurugan, Macro Cenzato, Giovanni Broggi, Dezhi Kang, Kenichiro Kikuta, Yuanli Zhao, Hengzhu Zhang, Shinsuke Irie, Yuping Li, Boon Seng Liew, Yoko Kato

**Affiliations:** 1 Department of Neurosurgery, Second Affiliated Hospital of Soochow University, Suzhou, China; 2 Centre for Minimally Invasive Neurosurgery, Prince of Wales Private Hospital, Randwick, New South Wales Australia; 3 Center for Endoscopic and Minimally Invasive Neurosurgery, Stuttgart, Germany; 4 Department of Neurosurgery, National Defense Medical College, Saitama, Japan; 5 Department of Neurosurgery, School of Medicine, Kyungpook National University, Daegu, South Korea; 6 Department of Neurosurgery, Juha Hernesniemi International Center for Neurosurgery, Henan People’s Provincial Hospital, University of Zhengzhou, Zhengzhou, China; 7 Department of Neurosurgery, Apollo Speciality Hospital, Chennai, India; 8 Department of Neurosurgery, Niguarda Hospital, Milano, Italy; 9 Department of Neurosurgery, Fondazione IRCCS Istituto Neurologico Carlo Besta, Milan, Italy; 10 Department of Neurosurgery, First Affiliated Hospital of Fujian Medical University, Fuzhou, China; 11 Department of Neurosurgery, University of Fukui, Fukui, Japan; 12 Department of Neurosurgery, Beijing Tiantan Hospital Affiliated to Capital Medical University, Beijing, China; 13 Department of Neurosurgery, Clinical Medical College of Yangzhou University, Yangzhou, China; 14 Department of Neurosurgery, Kushiro Kojinkai Memorial Hospital, Kushiro, Japan; 15 Department of Neurosurgery, Hospital Sungai Buloh, Sungai Buloh, Malaysia; 16 Department of Neurosurgery, Fujita Health University, Toyoake, Japan

**Keywords:** Keyhole approach, Neurosurgery, Brain tumor, Aneurysm, Expert concensus

## Introduction

Microneurosurgery made its debut in the early 1960s. It became popular quickly in the medical field and soon became a primary method in neurosurgery, as it improved the efficacy of neurosurgery, reducing surgically related injuries. Over the past five decades, the accumulation of microsurgery experience, improvements in microsurgical techniques, refinement of microinstruments, and advanced preoperative diagnostic imaging have enabled the evolution of microneurosurgical techniques and the further reduction of surgery-related trauma. These advancements have made it possible for neurosurgeons to treat more complicated lesions via smaller craniotomies [35, 60]. Keyhole neurosurgery, a combination of modern microsurgical techniques, preoperative imaging, neuroendoscopy, and the modern concept of minimally invasive surgery, is a technique representing the medical advances from microneurosurgery to minimally invasive neurosurgery [19, 63, 88].

Keyhole neurosurgery is a new, minimally invasive concept of microsurgery based on tailored, targeted, and direct microneurosurgical techniques [26, 70]. With specific anatomic and pathological structures and utilizing the “keyhole effect”, this method is designed precisely as an individualized approach to minimize the anatomic window and expose the lesions adequately, thus reduces unnecessary exploration of the surgical site and brain retraction. The “keyhole” concept involves not only smaller exposures, but also fewer surgery-related complications, the main and more important goal [62]. Pre-calculation of the size of the incision, bone window, and location of the craniotomy is based on the need for only a small dura opening with less brain exposure and retraction and is done in accordance with the principle of “maximal surgical efficiency while minimizing approach-related injury [10,19,30,64].” In general, a 4-cm incision and a cranial bone window of about 2.5 cm in diameter are applied wieldy in the keyhole surgery for deep lesions [74, 79, 87].

Keyhole microsurgery optimizes the cosmetic outcomes. This fact, along with the reduction in surgery-related complications, which improves patient’s acceptance of the surgery. In addition, surgeons can spend more time and focus on treating the lesions, thus achieving maximal surgical effects, shortening hospitalization times, and reducing treatment costs [49, 61, 88].

It has become clear to many of us that despite a growing literature supporting the use of selected keyhole approaches for certain intracranial pathologies, the literature lacks on well-established and standardized practices in this field. Given the importance of developing a collection of standard practices and acceptable indications for these relatively new approaches, we felt that an expert description was timely and important for both guidance about best practices, medicolegal protection, and for defining terms so the literature could be more clearly elaborated in the future.

To this end, the International Society on Minimally Invasive Neurosurgery (ISMIN) assembled organized experts in keyhole and minimally invasive surgery in Suzhou, China, in November 2018 from all over the world to compile a summary of the literature and expert opinions and guidance into a single statement. That is intended to improve neurosurgeons’ knowledge of keyhole microsurgical technology, to standardize the operative procedures, and to promote the development of minimally invasive neurosurgery all over the world.

## Methods

### Definitions of levels of recommendation

The concept of linking evidence to recommendations has been further formalized by the American Association of Neurological Surgeons (AANS) and the Congress of Neurological Surgeons (CNS). Our consensus followed this to report medical evidence and to refer to this guideline:

- Class I evidence, Level I recommendation, defined as evidence from one or more well-designed, randomized controlled clinical trial, including overviews of such trials;
- Class II evidence, Level II recommendation, defined as evidence from one or more well designed comparative clinical studies, such as non-randomized cohort studies, case-control studies, and other comparable studies, including less well-designed randomized controlled trials;
- Class III evidence, Level III recommendation, defined as evidence from case series, comparative studies with historical controls, case reports, and expert opinion, as well as significantly flawed randomized controlled trials. A summary of these categories of evidence can be viewed at https://www.cns.org/guidelines/guideline-procedures-policies/guideline-development-methodology

### Basic philosophical approach towards the development of this statement

We recognized from the onset that the development of Level I or II recommendations was almost certainly impossible given the complete absence of randomized controlled trials in the field which many of us suspect that a well-done trial definitively answering these questions is unlikely to occur (what patient will sign up for the bigger than necessary vs minimum necessary craniotomy trial just to prove this point?). Thus, it is important to note that we did not aim to create a set of formal guidelines on this topic on the level of a joint AANS/CNS statement, and thus, we want to be clear that we aimed to provide a single summary of the state of the art in keyhole brain surgery at the time of this paper.

### Meeting of experts and drafting the statement

Members of the expert panel met in Suzhou, China, November 5, 2018, as a subcommittee meeting at the 2018 Education Course of the ISMIN. The meeting utilized the outlines created from the literature review as a draft for structuring the discussion. Opinions of the experts were transcribed and discussed in detail to ensure that the statement best reflects the experts’ view.

## Findings of the committee based on literature and expert opinions

### Commonly applied keyhole microsurgery approaches

#### Supraorbital keyhole approach

*Indication (Table* *1**)*

**Table 1:** Indications of supraorbital keyhole approach

Indication within diseases	Level of recommendation
Anterior communicating artery aneurysms	Level II [53, 79]; Level III [7, 11, 23, 29, 31, 35, 38, 55–59, 62, 75, 77, 87]
Internal carotid artery aneurysms	Level II [53, 79]; Level III [11, 23, 35, 38, 55, 56, 58, 59, 77, 87]
Middle cerebral artery aneurysms Posterior communicating artery aneurysms	Level II [53, 79]; Level III [11, 13, 23, 35, 38, 55–59, 77, 87] Level II [53, 79]; Level III [7, 23, 29, 35, 38, 55–57, 59, 77, 87]
Basilar artery aneurysms	Level III [37, 38, 77, 87]
Posterior cerebral circulation aneurysms	Level III [35, 37, 38, 77, 87]
Ophthalmic artery aneurysms	Level III [7, 23, 35, 38, 56, 59, 77, 87]
Proximal superior cerebellar artery aneurysms	Level III [38, 77, 87]
Anterior cerebral artery aneurysms *ACA A1 and A2 proximal aneurysms*	Level III [35, 38, 57, 59, 77, 87] Level III [35, 38, 57]
Anterior choroidal artery aneurysms	Level III [35, 38, 56, 57, 59, 77, 87]
Pituitary adenoma	Level II [79]; Level III [14, 41, 62]
Tuberculum sellae meningioma	Level III [14, 25, 26, 52, 62, 74, 80]
Olfactory groove meningioma Fronto-basal region meningioma	Level III [25, 26, 62, 74, 80] Level III [25, 41, 64]
Suprasellar craniopharyngioma	Level II [79]; Level III [6, 14, 41, 52, 62, 80]

Lesions located around the sellar region and central skull base are suitable for application of supraorbital keyhole approach.

*Exposure zones (Table* *2**)*

**Table 2:** Exposure zones of supraorbital keyhole approach

Anterior cranial fossa	Anterior circulation artery	Posterior circulation artery
The frontal lobe base	Anterior communicating artery	Posterior clinoid process
Anterior clinoid process	Anterior cerebral artery (*A1 and A2 proximal*)	Basilar artery apex
Optic canal	Internal carotid artery	Posterior cerebral artery (*P1 segment*)
Olfactory sulcus	Middle cerebral artery (*M1 and M2 segments, part of M3 segment*)	Superior cerebellar artery proximal
Olfactory tract	Anterior choroidal artery	
Optic nerve	Posterior communicating artery	
Optic chiasm	The contralateral carotid artery medial surface	
Oculomotor nerve	Anterior cerebral artery A1 and A2 proximal	
Pituitary stalk	Middle cerebral artery M1 and M2 proximal	
Diaphragm sellae		
Dorsum sellae		
Anteromedial temporal lobe		
Anterior upper pontine		
Interpeduncular cistern		

*Head position*

With the patient in a supine position, the Mayfield head holder is applied to fix the patient’s head. The head is rotated about 10°–60° to the contralateral side depending on the site and size of the lesion. The head position could be modified according to the requirement of the exposure areas.

*Surgical procedures*

- A 3–4-cm skin incision is made within the eyebrow, and the subcutaneous and frontal fascia are separated. The frontalis muscle is cut parallel to the frontal skull base approximately 1 cm above its insertion into the orbicularis muscle, and the temporalis fascia is cut along the superior temporal line for approximately 2 cm. The frontal periosteum is incised in a semicircle shape from the superior temporal line, and then stripped and flipped. The base is located in the superior orbital rim. The temporalis muscle is bluntly separated, pushed 1.0–1.5 cm behind the superior temporal line.
- A bone hole of about 3–5 mm diameter behind the frontal bone zygomatic process (keyhole position) is milled out. The milling cutter runs along the orbital roof from the hole and back to create a bone flap of approximately 2.0 cm × 2.5 cm. The dura is opened in a flap shaped with the base at the orbital rim. The frontal lobe is gently lifted from the base using a paddy or brain retractor and cerebrospinal fluid (CSF) constantly absorbed, aiming to decrease the intracranial pressure.
- When finishing the intracranial procedure, the dura should be tightly sutured without a drainage tube. A skull lock or connector is used to fix the bone flap. The periosteum and myofascia are sutured layer by layer. The skin flap can be closed with continuously intradermal absorbable stiches or using metal cosmetic sutures, surgical stapler, to reduce scar.
  *Key points for recommendations*

The skin incision is placed within an eyebrow for pleasing cosmetic outcome.

The head is extended about 20°–25°, so that the frontal lobe moves backward because of gravity and leaves the anterior skull base to reduce intraoperative traction.

Slightly rotation of the head about 5°–15° to the contralateral side is helpful for the contralateral approach surgery, and it also offers the surgeon a more comfortable operation direction.

Attention should be focused on protecting the supraorbital nerve to avoid the risk of frontal numbness. The bone hole could be lateral to the superior temporal line, and the location may not be too low to avoid milling through the orbital wall.

When designing the bone window, we should avoid opening the frontal sinus. However, when this occurs, a tight repair is needed.

The inner plate of the supraorbital bone window edge is drilled out to gain greater visual space. Some bone ridge protrusions in the anterior skull base could be extradurally removed to increase the volume of the surgical corridor.

After opening the dura mater, the skull base is explored further to expose and open the chiasmatic and carotid artery cisterns to further release CSF, relaxing the frontal lobe and providing an optimal trajectory towards the skull base.

Endoscope and tube-shaft instruments can improve visualization and are particularly useful in selected cases, such as low-lying olfactory groove meningiomas, in which the lesion is not entirely within the surgeon’s direct line of sight.

It is recommended that the incision area could be compressed for several minutes after closing the skin flap for hemostasis, so as to reduce the incidence of subcutaneous hemorrhage and swelling.

The anterior clinoid process or posterior clinoid process may be drilled to provide a wide manipulation space for clipping of basilar artery aneurysms, if necessary.

In this approach, there are few venous effects on surgical access. Sometimes, we need to dissect sylvian fissure to reduce the traction between frontal and temporal lobes, or to increase the exposure of the internal part of sylvian fissure and temporal lobe.

*Limitations (Table* *3**)*

**Table 3:** Limitations and solutions of supraorbital keyhole approach

Limitations	Solutions
Limited exposure view and narrow surgical corridor	Reducing intraoperative ICP by draining CSF to increase the intracranial operating space (*Level III* [23, 35, 38, 64]).
	Preoperative lumbar puncture or ventricular drainage (*Level III* [35, 38])
	The neuronavigation system (*Level III* [62, 64]) Endoscopic-assisted keyhole surgery (*Level III* [23, 26, 30, 38, 62, 64])
Limited microinstruments	The invention of special keyhole-adapted microinstruments could solve these problems, including Gun-type rod-shaped, slim and tube shaft-designed tools (*Level III* [62, 64])
Postoperative palsy	Percutaneous mapping of the frontal branch of the facial nerve (*Level III* [58]). Small skin incision, respecting the anatomical pathway of the supraorbital nerve route (*Level III* [23, 26])
Unfamiliar with keyhole surgery	Performing anatomic dissection practice, and trained under the supervision of experienced senior neurosurgeons (*Level III* [64])
Influence on the appearance of eyebrow	Meticulous wound closure, particularly of the brow skin incision. Closing the skin layer with a running subcuticular stitch (e.g., 5-0 Prolene, Prolene, noninvasive metal sutures) without any suture knots (*Level III* [35, 52]). The tape can be used to further close the skin incision. If patient with light eyebrows, the eyebrow incision should be avoided.

#### Pterional keyhole approach

*Indication (Table* *4**)*

**Table 4:** Indication of pterional keyhole approach

Indication within diseases	Level of recommendation
Anterior Communicating Artery Aneurysms	Level II [16] Level III [7, 12, 38, 72, 81]
Internal carotid artery aneurysms	Level II [16] Level III [12, 38, 72, 75, 81]
Middle cerebral artery aneurysms	Level II [16] Level III [12, 20, 29, 35, 38, 47, 48, 72, 75, 81]
Posterior communicating artery aneurysms	Level III [7, 20, 29, 35, 37, 38]
Ophthalmic artery aneurysms	Level III [20, 38]
Posterior cerebral artery aneurysms	Level III [38]
Anterior choroidal artery aneurysms	Level III [12, 38]
Sphenoid wing meningiomas	Level III [5, 76]
Parasellar meningiomas	Level III [76]

The pterional keyhole approach provides early visualization of the optic nerves and anterior circulation vessels while minimizing exposure and risk to unneeded parts of the frontal lobe and temporal lobe. In addition, selected patients with posterior circulation aneurysms, tumors around sellar region, and sphenoid wing meningiomas are suitable for application of pterional keyhole approach.

*Exposure zones (Table* *5**)*

**Table 5:** Exposure zones of pterional keyhole approach

Anterior cranial fossa	Anterior circulation artery	Posterior circulation artery
Suprasellar region	Ipsilateral posterior communicating artery	Basilar artery apex
Parasellar region	Anterior choroidal artery	Superior cerebellar artery proximal
Retrosellar region	Internal carotid artery	Posterior cerebral artery (*P1 and P2a*)
Cavernous sinus superior wall	Ophthalmic artery	
Frontal part of the lateral wall of the cavernous sinus	Middle cerebral artery (*MCA M1–M4 segment*)	
Sphenoid ridge	Ipsilateral and contralateral anterior cerebral artery	
Temporal pole	(*A1 and A2 proximal*)	
Frontal pole Anterior cranial fossa	Contralateral MCA M1 and M2 proximal The medial and bifurcation part of internal carotid artery	
Front end of the middle cranial fossa		
Interpeduncular cistern		
Prepontine cistern		

*Head position*

The patient’s head is leaned slightly back so that the frontal lobe inclines and leaves the orbital roof by gravity; the head is rotated about 30°–60° to the contralateral side based on actual need: a greater rotation angle to the contralateral side is needed when the lesion is closer to the frontal end. The head is tilted slightly about 15° to the contralateral side to compensate for the upward inclination angle along the middle skull base.

*Surgical procedures*

- About 2 cm outside the keyhole (posterior to the temporal line at the level of frontal skull base), around the pterion, an anterior hairline incision about 4–5 cm long is made. The subcutaneous tissue and temporal fascia are incised parallel to the skin incision. The temporal muscle is incised along the direction of the muscle fibers through the pterion and expanded with the mastoid expander. An osseous depression may be seen in the exposed central skull bone, which is the mark of the sphenoid ridge on the surface of the skull. A bone hole about 3-5 mm diameter is drilled on the bottom of the sphenoid ridge, and from this location, a bone flap about 2.5 cm in diameter is milled out, and one- to two-thirds of the lateral sphenoid ridge is removed; if necessary, the edge can reach the lateral side of the supraorbital fissure. Centering on the sphenoid ridge, the dural flap is cut open and retracted forward to expose and then open the sylvian fissure.
- Closure is basically as same as that for the supraorbital approach. The hairline incision can be sutured in a routine manner.

*Key points for recommendations*

Usually, the exposure area could be adjusted according to surgery requirements by moving up or down the incision location, thus the location of the bone window and exposure area of the frontal and temporal lobes.

For anterior communicating artery aneurysms, the sylvian fissure is usually located at the lateral third of the bone window; two-thirds of the brain tissue exposed under the bone window is the frontal lobe, and the remaining third is the temporal lobe.

For middle cerebral artery aneurysms or lateral posterior communicating artery aneurysms, the sylvian fissure is usually placed in the center of the bone window so that the temporal lobe can be slightly retracted after opening the sylvian fissure.

For the aneurysm pointing towards the dorsal part of the parent artery, the pterional keyhole approach was more favorable for exposing the aneurysm neck.

While clipping the anterior communicating artery aneurysm via the pterional keyhole approach, olfactory nerve damage caused by raising the frontal base could be reduced through this lateral approach.

For middle cerebral artery aneurysms, the pterional keyhole approach could be used to evacuate hematoma in the temporal lobe simultaneously.

If necessary, the terminal part of the sylvian vein converge into the sphenoparietal sinus could be cut in order to retract the temporal lobe.

*Limitations (Table* *6**)*

**Table 6:** Limitations of pterional keyhole approach

Limitations	Solutions
Surgical view is obstructed by Sylvian vein	Dissecting the Sylvian fissure, placing the Sylvian vein behind the brain retractor and retracing the temporal lobe, opening the carotid cisterns to release CSF (*Level II* [16]; *Level III* [7, 12, 81]).
Narrow surgical corridor	The sphenoid ridge is removed with a diamond drill to provide wide manipulation space of the skull base area. The sphenoid ridge is the most important landmark to precisely position the scheduled keyhole mini-craniotomy (*Level III* [20, 48, 72]). Introducing neuroendoscope (*Level III* [12])
Postoperative temporal muscle atrophy	Making a vertical incision along the muscle fibers on the temporal muscle, or dissecting the temporal muscle by the retrograde dissection method. Minimal preparation and dissection of bones and muscles can decrease iatrogenic surgical trauma, cranial deformities and temporal muscle atrophy significantly (*Level II* [16] *Level III* [12]).
Injury to the temporal branch of the facial nerve	The incision of pterional keyhole approach is made between the branches of the facial nerve. Cutting the temporal muscle near the edge of its insertion to the temporal bone. Applying the interfascial or subfascial technique (*Level II* [16]; *Level III* [12, 47, 48, 76, 81]).

#### Subtemporal keyhole approach

*Indication (Table* *7**)*

**Table 7:** Indications of subtemporal keyhole approach

Indication within diseases	Level of recommendation
Tumors confined in the petroclival and suprasellar region	Level III [36, 60, 73]
*Hypothalamic gliomas* *Retrochiasmatic craniopharyngiomas* *Trigeminal neuromas* *Pituitary adenomas* *Petroclival meningiomas* *Pontine cavernous hemangioma*	
Posterior cerebral artery (P2-P3 segment) aneurysms	Level III [35, 37, 60]
Middle fossa arachnoid cysts	Level III [39, 66]

Lesions located within the petroclival and suprasellar region are suitable for application of subtemporal keyhole approach. In addition, the subtemporal keyhole approach can be applied to some posterior cerebral circulation aneurysms

*Exposure zones (Table* *8**)*

**Table 8:** Exposure zones of subtemporal keyhole approach

Super- and parasellar	Brain	Posterior fossa
Temporobasal skull base Anterior clinoid process	Anterolateral midbrain Anterolateral pons	Tentorium Upper clivus
Posterior clinoid process	Anterosuperior cerebellar	Superior cerebellar artery
Internal carotid artery		Basilar artery
Posterior communicating artery		Posterior cerebral artery
Anterior choroidal artery		CN V, CN VI, CN VII, CN VIII
Pituitary stalk		
CN II, CN III, CN IV		
Optic tract		
Tentorium		

*Head position*

The patient is laid in the supine position with shoulders elevated, and the head is rotated about 90° to the contralateral side of the lesion, keeping the zygomatic arch in the horizontal position. The head is extended back about 15° so that the trachea is not oppressed; the head is lateroflected 15° to compensate for the upward incline angle along the middle skull base. This head position can tilt the temporal lobe away from the skull base by gravity.

*Surgical procedures*

A skin incision is made about 1 cm in front of the tragus. Starting from the superior zygomatic arch, a vertical incision approximately 4 cm above the zygomatic arch is made. A Y-shaped temporalis fascia incision is made and retracted to expose the surgical field. The temporal muscle is incised longitudinally and retracted. A hole is drilled posterosuperior to the zygomatic arch base, and a bone window about 2.0–2.5 cm in diameter is created using the milling cutter. The dura is incised in a flap shape and flipped inferiorly. The temporal base is lifted gently to gradually release CSF to reduce the intracranial pressure (ICP). The edge of the tentorial incisura is exposed gradually deeper in.

*Key points for recommendations*

The subcutaneous tissue is separated, making sure to avoid damaging the frontal branch of the facial nerve and superficial temporal artery.

Individualized keyhole approaches for posterior cerebral circulation artery aneurysms are safe and effective.

Incision of the tentorium could further resect lesions in the posterior fossa, and the petrous apex can be removed with a diamond drill to provide enough space to deal with the lesions in the petroclival region. Generally, the surgical manipulating corridor is limited to the top of the internal auditory canal.

A preoperative lumbar drain may be helpful, since access to the CSF spaces will be able only when reaching the tentorial edge.

Although the Labbe' vein is not visible directly below the bone window, it may be damaged by traction when lifting the base of the temple. If necessary, the arachnoid membrane on the venous surface can be separated to increase the degree of dissociation and make the operation space larger.

*Limitations (Table* *9**)*

**Table 9:** Limitations and solutions of subtemporal keyhole approach

Limitations	Solutions
Deep-seated location and limited manipulating surgical corridor	Releasing CSF and reducing ICP through cisterna ambiens opening (*Level III* [36, 60]) Lumbar drainage (*Level III* [60])
	Introducing neuroendoscope (*Level III* [39, 66, 73])
Insufficient exposure of lesions in posterior cranial fossa The tentorial sinus affects the incision of the tentorium	The tentorium is subsequently divided, lengthening the incision. Opening the Meckel’s cave to reach the internal auditory canal (*Level III* [60, 73]) A tentorial incision perpendicular to the superior petrosal sinus is made by coagulation or clipping. Hemostasis with electrocoagulation and hemostatic materials packing (*Level III* [60, 73])
Injury of the trochlear nerve	Dissecting the cerebellar tentorial segment of the trochlear nerve, and then cut the tentorium cerebelli to enlarge the surgical manipulating corridor (*Level III* [60])
Postoperative temporal atrophy	Making a short vertical incision at the posterior margin of the temporal muscle (*Level III* [60]).

#### Median suboccipital keyhole approach

*Indication (Table* *10**)*

**Table 10:** Indication of median suboccipital keyhole approach

Indication with diseases	Level of recommendation
Posterior inferior cerebellar arteries aneurysm	
Posterior cranial fossa tumors	Level III [36]
*Medulloblastoma*	
*Ependymoma*	
*Capilliary hemangioblastoma*	
*Cerebellar metastases*	
Brainstem glioma	Level III [65]

The median suboccipital keyhole approach is appropriate for lesions located in the cerebellar vermis, fourth ventricle, dorsum of pons, and medullary dorsal parts *(e.g., Distal posterior inferior cerebellar artery (PICA) aneurysms).*

*Exposure zones*

The posterior circumference of the foramen magnum, marginal sinus, occipital sinus, the cerebellar tonsil and inferior vermis, cervicomedullary junction, posterior inferior cerebellar artery, and the fourth ventricle, pons and medullary dorsal parts.

*Head position*

The patient is laid in a prone position and the head flexed forward to fully extend the craniocervical junction with the tentorium in a perpendicular plane.

*Surgical procedures*

An upward suboccipital median incision from 1 cm below the foramen magnum and approximately 4-cm long is made. The scalp is incised and separated sharply along the midline. A bone window about 2.5 cm in diameter is created from the trailing edges of the foramen magnum in the upward direction. The dura is incised in a X or Y shape and the occipital sinus closed with cautery or suture. The cisterna magna is opened, the cerebellar tonsil lifted, the arachnoid adhesion separated, and the fourth ventricle exposed through the cerebellomedullary fissure approach.

*Key points for recommendations*

This approach can clearly expose the entire fourth ventricle from the foramen magnum to the lower aqueduct. Dissecting more laterally within the ventricular chamber, the vestibular area and the foramen of Luschka of the fourth ventricle can be observed.

The procedures of the suboccipital “open-door” keyhole craniotomy require two paramedian bone holes and removal of the internal occipital crest.

Stages of craniotomy: (1) two paramedian burr hole trephinations; (2) median suboccipital craniotomy; (3) Due to partial removal of the posterior arch of the atlas without laminectomy, the exploration of the cervicomedullary junction can be extended.

Distal PICA aneurysm clipping via median suboccipital keyhole approach could obtain satisfactory results.

The occipital sinus should be treated with sutures or bipolar coagulation. It is usually necessary to repair the dura to prevent cerebrospinal fluid leakage.

*Limitations (Table* *11**)*

**Table 11:** Limitations and solutions of median suboccipital keyhole approach

Limitations	Solutions
Cerebrospinal fluid leakage	The dural opening should be closed watertight using interrupted or running sutures (*Level III* [36]).
Limited surgical view from inferior to superior	Drainage of CSF from cistern magna, cerebellomedullary, and prepontine cisterns aids in relaxation of cerebellum and obtaining adequate exposure (*Level III* [36]). Careful preoperative positioning of patients and modifying the position during the operation, which provide an excellent overview of the target (*Level III* [36])
Hydrocephalus	Endoscopic third ventriculostomy can be performed prior to tumor resection as tumor removal could be proposed as an effective method for reducing the probability of postoperative shunting (*Level III* [65])
Limited visualization of fourth ventricle	After the arachnoid membrane is opened sufficiently and the cerebellum is relaxed well, the cerebellar tonsils can be retracted bilaterally, and the inferior cerebellar vermis can be easily elevated upward to expose the whole fourth ventricle region (*Level III* [36]).

#### Retrosigmoid keyhole approach

*Indication: (Table* *12**)*

**Table 12:** Indication of retrosigmoid keyhole approach

Indication within diseases	Level of recommendation
Hypoglossal neurinoma Vestibular schwannomas Facial nerve neuromas Cochlear nerve neuromas Petroclival, tentorial, CPA meningiomas	Level III [3] Level III [15, 24, 36, 40, 43, 51] Level III [43] Level III [43, 51] Level III [36, 40, 51]
Deep pontine lesion (glioma, cavernous angioma etc.)	Level III [36, 54]
CPA Cholesteatoma	Level III [36]
MVD of HFS* *trigeminal neuralgia* *glossopharyngeal neuralgia*	Level III [10, 42, 67]

*MVD* Microvascular decompression, *HFS* hemifacial spasm

The retrosigmoid keyhole approach provides a safe and effective route to the cerebellopontine angle, upper and middle clivus, and with minor variation may provide an avenue to the lower clivus and foramen magnum.

*Exposure zones*

The retrosigmoid keyhole approach can expose the following anatomic structures: the trigeminal nerve, facial nerve, acoustic nerve, lower cranial nerves, lateral and anterior lateral pons, lateral cerebellar hemisphere, vertebral artery, and posterior inferior cerebellar artery. For treating lower cranial nerve lesions, the surgical incision and bone window position may descend accordingly.

*Head position*

The patient is placed in the lateral park bench position. The patient’s head is rotated about 10°–20° to the contralateral side from the lateral position to provide a direct view angle with less retraction to the cerebellar hemisphere and can open the cerebellopontine angle cistern successfully. During the operation, based on the location of the lesion, the view angle can be adjusted by changing the degree of inclination (left and right) of the operating bed.

*Surgical procedures*

Starting at the junction line of the external occipital protuberance and mastoid roots, about 1.5–2.0 cm posterior to the mastoid roots, a vertical, oblique (along the hairline) incision, or a horizontal incision of about 4 cm long is made. In the case of craniectomy, a burr hole about 2.5 cm in diameter is created behind the mastoid. In the case of craniotomy, a burr hole of about 5 mm is made at the junction between the transverse and the sigmoid sinus junction. Then, a craniotomy of about 2.5 cm in diameter is performed using a craniotome. The top of the bone window is near the horizontal inferior margin of the transverse sinus, and its lateral edge is on the posterior edge of the sigmoid sinus. Mastoid air cells are closed with bone wax. The dura is incised in a flap shape with its base located at the sigmoid sinus. The lower lateral cerebellum is gently lifted from the petrous bone, and the lateral cerebellomedullar cistern is opened gradually to release CSF. The cerebellopontine angle cistern and lateral cerebellomedullar cistern are dissected to expose the anatomical structures.

*Key points for recommendations*

Removal of the anterior inner edge of the craniectomy over the sigmoid sinus can significantly increase the angle for visualization.

Watertight dural closure and sealing of mastoid air cells are important to prevent postoperative CSF leakage.

The petrous vein should be preserved as far as possible to prevent cerebellum or brain stem swelling caused by dysfunction of venous drainage.

Neuroendoscope could help recognize hidden and deep-lying structures like small arteries and veins responsible for vascular compression on the surface of cranial nerves.

Diamond drill could be used to remove the posterior wall of the internal auditory canal to resect the tumor inside. Introducing the neuroendoscopy could clear observe the internal auditory canal and reduce the opening area of the internal auditory canal.

*Limitations (Table* *13**)*

**Table 13:** Limitations and solutions of retrosigmoid keyhole approach

Limitations	Solutions
CSF leakage	Watertight dural closure and packing of mastoid air cells using bone wax, glue or free fascial tissue. Repairing the defect of internal acoustic meatus with muscle mixed glue and gelfoam sponge graft. Perform lumbar CSF drainage and compression on the region of the operative wound (*Level III* [15, 36]).
High ICP	Continuous drainage of cerebrospinal fluid after release the CSF by lumbar cistern drainage during operation. Release CSF by opening the lateral cerebellomedullary cistern (*Level III* [15, 36])
Obstructed surgical view by nerve and vessels	Introducing neuroendoscope or modifying the view angle of microscope during the operation (*Level III* [40, 42])
Surgery-related cranial nerve injury	Intraoperative electrophysiological monitoring (*Level III* [15, 54])

#### Interhemispheric transcallosal keyhole approach

*Indication (Table* *14**)*

**Table 14:** Indication of interhemispheric keyhole approach

Indication within diseases	Level of recommendation
Lateral ventricular tumors	Level III [1]
Third ventricular tumors	Level III [9, 28, 34]
Interhemispheric transcallosal hemispherotomy	Level III [8]
Anterior cerebral artery aneurysms (A2, A3)	Level III [19, 35, 84]
Anterior communicating artery aneurysms	Level III [19]

The interhemispheric keyhole approach has been applied for the lesions between the medial side of the brain and cerebral falx, the corpus callosum, the central lateral ventricle, the anterior cerebral artery along the surface of the corpus callosum, and the suprasellar region.

*Exposure zones*

The interhemispheric transcallosal keyhole approach can expose the following anatomic structures: corpus callosum, cerebral falx, the distal segment of the anterior cerebral artery, body of the lateral ventricle, third ventricle, and thalamus.

*Head position*

The patient is placed in supine position, and the head is flexed approximately 30° to 45° angle with the horizontal line to allow the surgical dissection near to the perpendicular plane.

*Surgical procedures*

A linear incision about 4-cm long is made 1 cm beside and parallel to the midline or perpendicular to it with two thirds of the incision on the side of the craniotomy. The location of the lesion determines the specific incision points. A burr hole about 3–5 mm in diameter is drilled immediately beside the midline, and a small bone flap about 2–2.5 cm is milled out to expose the sagittal sinus at one edge of the craniotomy. The dura is opened in a curvilinear fashion with base towards the midline. The head is rotated 10° to 30° and lateroflected to the craniotomy side, tilting the hemisphere away from the midline. The arachnoid membrane near the sagittal sinus is separated carefully without damaging the large draining veins. If necessary, a section of the vein is freed up by separating the arachnoid on its surface to increase the degree of displacement. Dissection is performed along the lateral cerebral hemisphere descending gradually to the corpus callosum, which is incised longitudinally for about 1.5–2 cm to reach the lateral ventricle. Caution has to be taken to avoid injure of the pericallosal arteries.

*Key points for recommendations*

This approach can provide a direct midline orientation with symmetrical access to both lateral ventricles and both walls of the third ventricle.

The contralateral interhemispheric transcallosal approach can effectively expose the lesions located in the lateral part of lateral ventricle.

With this approach, the third ventricular chamber can be approached via an interforniceal, subchoroidal, transchoroidal, or transforaminal path.

Place the bone window to anterior coronal suture could avoid larger bridging veins through surgical corridor. A 1.5- to 2.0-cm callosal incision is beneficial to avoid the occurrence of permanent interhemispheric disconnection.

Neuronavigation is helpful to localize and to plan a targeted and tailored interhemispheric approach

*Limitations (Table* *15**)*

**Table 15:** Limitations and solutions of interhemispheric transcallosal keyhole approach

Limitations	Solutions
Interhemispheric retraction	The patient's head is fixed in a horizontal position and tilted approximately 45°–60° with sufficient gravitational retraction of the hemisphere; applying the osmotic solutions or releasing cerebral spinal fluid to reduce the ICP (*Level III* [1, 34]) The contralateral approach may minimize the need for retraction of the dominant frontal lobe for treatment of dominant-hemisphere lesions. If required, a retractor is usually placed superiorly to gently lift the falx, which protects the ipsilateral hemisphere (*Level III* [1]).
Venous injury	Applying special instruments and precise navigation to place the skin incision and craniotomy in a position free of bridging veins for a precise craniotomy (*Level III* [1]) Small burr hole may help to limit the extent of brain retraction; full free of the bridging veins can prevent venous injuries effectively (*Level III* [34]).
Callosal incision	Only for small tumors, and the length of the callosal incision should be limited to 1.5 cm (*Level III* [34])
Limited operation space or high ICP	Releasing cerebral spinal fluid (*Level III* [34, 84]) The angled endoscopes can be helpful to allow a panoramic view of the resection cavity and to avoid leaving residual tumor behind (*Level III* [28]).
Intraoperative bleeding	Bipolar coagulation on the walls of the ventricle is minimized to prevent scarring, and most bleeding stopped with the application of small pieces of Surgicel (*Level III* [1])
Limited lateral exposure	Only performing for lesions located closer to the midline (*Level III* [34]) Using contralateral approach to better expose the contralateral deep structures (*Level III* [34])

#### Infratentorial supracerebellar keyhole approach

*Indication (Table* *16**)*

**Table 16:** Indications of infratentorial supracerebellar keyhole approach

Indication within diseases	Level of recommendation
Pineal region tumors *Pinealomas* *Cholesteatoma* *Germinomas* *Glioblastoma multiforme* *Pineocytoma* *Pineoblastoma* *Medulloblastoma*	Level III [4, 9, 33, 34, 71, 82]
Meningiomas of pineal region Meningiomas of the lateral ventricles	Level III [4] Level III [44]
Tumors in the posterior part of third ventricular	Level III [4, 34]

The infratentorial supracerebellar keyhole approach is a common strategy used to access midline and paramedian lesions located beneath the deep venous system in the pineal-tectal region.

*Exposure zones (Table* *17**)*

**Table 17:** Exposure zones of infratentorial supracerebellar keyhole approach

Supracerebellar corridor	Pineal region
Confluence of sinuses	Splenium of the corpus callosum
Inferior surface of the tentorium	Pineal gland
Straight sinus	Posterior commissure
Upper vermis	Pulvinar thalami
Tentorial cerebellar surface	Crus fornicis
Central cerebellar vein	Hippocampal commissure
Great cerebral vein of Galen	Velum interpositum

*Head position*

1. The semi-sitting position: The patient is placed in the semi-sitting position with slight flexion of the neck. Although this position is associated with a risk for venous air embolism, it may provide maximal relaxation of the cerebellum, with limited use of cerebellar retraction and taking advantage of the effect of gravity.
2. The concorde position: (A) Surgeon standing at the head of the patient: The patient is placed in prone position. Then, the upper body is lifted or raised around 15°–20° to promote appropriate venous circulation. Additionally, the head is flexed down to the external occipital protuberance-eyebrow line, which lies perpendicular to the floor. (B) Surgeon standing on the lateral side of the patient: The head could be anteroflected about 45° to bring the tentorium in a perpendicular plane. The neck flexed 30°–45° to the contralateral side, and the head tilted to the operative side 30°.

*Surgical procedures*

A 4-cm straight vertical incision is made from 5 mm above the external occipital protuberance in the midline or paramedian for the “paramedian infratentorial supracerebellar keyhole approach” (PISKA). A craniectomy or craniotomy located just inferior to the transverse sinus is carefully performed in keyhole fashion using a high-speed drill. Care has to be taken to protect the adjacent sinuses.

The dura is opened in a U-shaped fashion and reflected superiorly to expose the interface between the cerebellum and the tentorium. The positioning and the effect of gravity could be taken advantage of to obtain the maximum operating room [22, 32]. The draining veins should be preserved as far as possible in case of edema and venous infarction of the cerebellum [27, 68]. Some studies reported that the paramedian approach may allow working around the bridging veins to preserve them [27]. It is better to sacrifice smaller thin walled veins and preserve large veins, keeping in mind that there it is difficult to predict which vein, when occluded, will result in serious complication [32, 45].

The arachnoid overlying the vein of Galen is carefully dissected from lateral to medial. The basal veins of Rosenthal can be seen close to the tentorial notch, on both sides. Great care is needed at this point to avoid damage to the veins, since the basal veins of Rosenthal and the vein of Galen must always be preserved [22, 32, 68].

*Key points for recommendations*

In cases of lesions located lateral to the pineal region, we could apply a surgical corridor between the basal vein of Rosenthal and the tentorial edge. The dissection comes from lateral to medial, dividing small feeders from the posterior choroidal and superior cerebellar arteries.

If necessary, the periosteal sheet of the external protuberance can be used for watertight closure.

Cerebellar swelling might be caused by incision of the precentral vein of the cerebellum, which led to dysfunction of venous drainage of the cerebellum. We could dissect arachnoid overlying the precentral vein of the cerebellum to make it nomadic so that the vein or part of its branches can be preserved.

*Limitations (Table* *18**)*

**Table 18:** Limitations and solutions of infratentorial supracerebellar keyhole approach

Limitations	Solutions
Limited exposure and visualization structures	The neuroendoscope can be introduce through limited surgical corridors (*Level III* [4, 44, 71, 82]). Relaxation of the cerebellum by releasing cerebrospinal fluid and sufficient gravitational retraction of the cerebellum (*Level III* [4, 34])*.* The tentorium can be incised to expose supratentorial tumors (*Level III* [44]). Minor bridging veins over the tentorium may be divided to allow further cerebellar slump (*Level III* [44]). If necessary, the superior cerebellar vein and draining veins coming from the surface of the cerebellum can be coagulated and cut without postoperative deficits to provide a wider exposure (*Level III* [22, 44]).
Cerebellar swelling or dysfunction of venous drainage	Preserving the normal veins as much as possible (dissecting arachnoid overlying the precentral vein of the cerebellum) (*Level III* [34])

### How to avoid complications

Small craniotomies do have some restrictions. The major limitations of keyhole surgery are as follows: the space for manipulations is limited, and the operational orientation is essentially fixed [63]. It is extremely difficult to modify the surgical corridor during an operation [62, 63]; for most aneurysm cases with intraoperative bleeding, the surgeon will control the bleeding and clip the aneurysm on his own, as space for the assistant to operate is limited [38, 62, 63]. Therefore, detailed preoperative planning plays the most important role in keyhole surgery.

### Treatment measures for intracranial hypertension

In cases of elevated intracranial pressure, the most effective measure is to open the brain basal cisterns to release CSF so as to create sufficient operating space [23, 35, 36, 38, 60, 64]. After the CSF is released, the brain tissue will retract on its own. Dehydrating agents (mannitol or hypertonic solutions) or an external ventricular drain may be used to decrease ICP [28, 35, 38]. In some lesions with a largely intracranial mass effect, releasing CSF during an early stage of the operation may be difficult, as the local brain cisterns have been compressed or disappeared. To solve this problem, preoperative lumbar puncture or lumbar subarachnoid drainage are options for intraoperative CSF release and reducing the ICP [15, 34, 38, 60] .

### Management of surgical incision

A skin incision within the eyebrows is relatively small in the supraorbital keyhole approach, but compared to the hairline incision, it is exposed and may seem not well- suited for patients with light eyebrows. Coagulation should be minimized during the approach to prevent tissue shrinkage. When closing the incision, the layers must be carefully matched and may be sutured subcutaneously with some noninvasive sutures [52, 62]. The subcutaneous tissue of the eyebrows is loose and prone to subcutaneous effusion. If this occurs, a pressure bandage or puncture drainage should be used immediately.

### Technique for intraoperative aneurysm rupture

The surgical procedures for aneurysm clipping using the keyhole approach are the same as those for the conventional microsurgical technique, even in the case of treating ruptured aneurysms. The same method is used to treat unexpected bleeding, and the senior surgeons performing keyhole surgery should be experienced in managing intraoperative premature rupture during aneurysm operations [7, 17, 53, 83].

In the case of severe bleeding due to the premature rupture of the aneurysm, two aspirators may be used to control the bleeding: the big one is used to aspirate the blood directly from the rupture site of the aneurysm, and the small one is used to remove the blood from the surgical field [35, 38, 46]. When the operating field is clear again, the parent artery can be dissected rapidly and occluded temporarily, or the neck of the aneurysm can be dissected directly, and then clipped, if possible.

### Prevention of CSF leakage

During the craniotomy in keyhole surgery, the first step is opening the cisterns to release CSF [35, 38]. Inadequate dural closure may lead to postoperative CSF leakage. Therefore, a watertight dural closure is essential to prevent postoperative CSF leakage [36]. When the keyhole surgery is performed at the skull base, the frontal sinus, mastoid air cells, petrous bone air cells, and anterior clinoid process air cells all may be opened. When openings occur, a thorough reconstruction of the skull base should be performed.

## Limitations and solutions in keyhole neurosurgery

### Limited exposure view and narrow surgical corridor

#### Effective intracranial operating space

The limited exposure and narrow surgical corridor may render keyhole surgery difficult. Inappropriate retraction of the brain due to limited operating space could cause severe surgery-related damage. For this reason, an important step is to decrease the ICP by releasing CSF from the basal cisterns and therefore increase the surgical space [23, 35, 38, 64].

When keyhole surgery is performed for lesions located in the skull base, it is critical to confine the craniotomy to the skull base. For example, in the supraorbital keyhole approach, the incision is hidden in the eyebrows, and the bone window reaches the superciliary arch; for the subtemporal keyhole approach, the temporalis is separated and distracted to both lateral sides to avoid the traction of the temporal muscle flap towards the temporal base in the conventional approach and to prevent the zygomatic arch blocking the surgical field. The keyhole approach avoids unnecessary structural damage and tissue exposure by retaining an effective operative space to meet the actual needs.

#### Automatic retractor assistance

As the keyhole microsurgery has such a narrow viewing angle and manipulation space, it is possible for only one surgeon to perform the operation. Therefore, an automatic retractor is a helpful surgical instrument for fixing the exposure zone without the aid of an assistant, plus it does not affect the surgical view. In particular, the automatic retractors that are fixed by bed-side support arms are more suitable for keyhole surgery, enabling easy adjustment of the flexible retractors. In most operations, only a short period of or even no retraction is necessary.

#### Precisely locating of the lesion

As the keyhole approach can reach the lesion from only one direction, and changing the surgical corridor during the procedure is extremely difficult, a precisely placed craniotomy and careful preoperative approach planning are preconditions for successful keyhole surgery [23, 35, 53, 63]. As many of the approached lesions have well-defined intracranial anatomical landmarks, locating tools such as neuronavigation are generally not needed for aneurysms, sellar tumors, cerebellopontine angle lesions, intraventricular lesions, and pineal region tumors.

But the neuronavigation system helps to find the precise position of small lesions in the brain parenchyma and estimate the progress of tumor resection, which could further reduce the unnecessary exploration of brain tissues [35, 62–64].

### Limited microinstruments

In the keyhole approach, it is difficult to work with the traditional microinstruments through the small craniotomy [9, 26, 63]. Therefore, the development of surgical instruments designed for keyhole surgery is important [35, 62, 87]. Recently, some novel microinstruments have been used for keyhole microsurgery, including a gun-like, rod-shaped instrument that can increase the visual space effectively during the operation and help avoid either the surgeons’ fingers or the instrument itself casting a shadow in the light beam of the microscope [9, 64, 79].

A variety of instruments can be used simultaneously in the keyhole approach, such as two brain spatulas, two aspirator tubes, bipolar forceps, or clip appliers. Furthermore, a neuroendoscope can be used to enhance visualization during the operation [42, 82, 87].

### Reduced field of view

The improvement of neuroendoscopes provides favorable intraoperative optical control in keyhole microsurgery, improving the light and sight of vision during an operation [4, 38–40, 44, 66, 71, 73, 80, 82]. A neuroendoscope with a diameter of 2–4 mm can provide bright light intensity, a clear depiction of the details of deep lesions, close-up imaging of the lesion, and extended viewing angles. The clear and extended view can avoid the traction of superficial structures, reducing the surgery-related injury to normal tissue and improving surgical orientation and safety.

### Less-experienced surgeons

Surgeons who are new to the field of keyhole surgery should not pursue a minimized bone window blindly, but rather focus on understanding the concept of minimal invasiveness in keyhole surgery, then make the bone window as small as possible. Initially, a larger bone window can be made, which is then reduced stepwise with the increasing experience of the neurosurgeon. Alternatively, junior surgeons may initially make a large incision with a small bone window. If unexpected events occur during the keyhole surgery, the bone window can be quickly expanded using the milling cutter and modified for the conventional craniotomy approach. Furthermore, it is suggested that junior surgeons should engage in anatomic dissection practice to familiarize themselves with minimally invasive operations and the indications for different keyhole approaches [79]. Further, it is suggested that junior surgeons be trained under the supervision of experienced senior neurosurgeons when first encountering the keyhole approach; such training will be very effective in improving the junior surgeons’ confidence and surgical safety techniques [64].

### Consideration of a craniotomy with a large bone flap

A larger bone flap may be considered for aneurysm patients with severe subarachnoid hemorrhage or a serious disturbance of consciousness, exposing them to a higher risk of postoperative cerebral vasospasm or intracranial hypertension [59]. If necessary, a decompressive craniectomy may be performed on such patients [38]. A larger craniotomy would also be a more appropriate approach for large tumors close to the surface or for arteriovenous malformations, as the whole lesion needs to be exposed. During these operations, a multi-angle approach may also be needed.

### Surgical strategy for large tumors

In terms of application of the keyhole approach for large tumors (>3 cm) located deeply in the brain, the keyhole approach has its own special amplification effect. This effect results in an increased surgical field with increasing distance from the approach entrance, meaning the deeper the intracranial location is, the larger the field of view that can be obtained through a small bone window by adjusting the angle of the microscope.

For large, deep-seated tumors, the standard surgical technique is to resect the tumors in a piece meal manner, as numerous important structures such as nerves and vessels surround the lesions. Therefore, a bone window of only 2 cm in diameter may well meet the basic requirements for such operations without increasing the tumor resection time. The surgical cavity provides a gradually increasing surgical space after partial tumor resection and allows the surrounding tumor tissues to drift into the central field of view, more likely achieving a complete tumor resection [26, 40].

Combining different keyhole approaches is a good method for treating large tumors involving multiple cranial fossae, thus avoiding a complex, extensive, single-approach operation with its associated significantly higher morbidity [85, 86]. Keyhole surgery for large tumors is individually designed according to the specific conditions.

### Preoperative imaging for keyhole surgery

Preoperative planning includes creating a precise three-dimensional (3D) concept of the surgical target area and the trajectory leading to it. This is achieved by the acquisition and study of detailed multimodality imaging series, including CT, MRI, f-MRI, diffusion tensor imaging–tractography, and detailed arterial and venous vascular imaging [2, 18, 21, 22, 48, 63, 69]. According to preoperative imaging, the direction of aneurysm and the compensation of collateral circulation are closely related to the choice of surgical approach. When aneurysm rupture with severe subarachnoid hemorrhage and increased ICP, narrow cistern are found, routine craniotomy is more suitable. If the tumor is large and the cistern is not easy to open, the application of lumbar cistern catheter before operation and the release of CSF during operation can effectively reduce ICP. When the bone structure in the surgical corridor affects the exposure of the lesion, it is necessary to distinguish the feasibility, advantages and disadvantages of resection of the related bone structure, or to use other approaches to avoid the obstruction of the bone structure. If necessary, a 3D-printed brain model can be constructed by multi-modal image fusion to verify the feasibility of keyhole surgery and increase surgical experience [38].

### Preparation and sterilization of the operation area

With a 4-cm skin incision in keyhole surgery, no hair shaving is necessary before operation. [35]. The shaved area can be limited to 1 cm around the incision, or the incision can be made on the hairline. Then, sterile drapes are applied to the operative area to isolate the surrounding hair.

### Craniotomy during the keyhole surgery

We suggest not to use perforators, but to practice, with a small drill, a little rectangular hole, oriented along the line of the predicted craniotomy, limited to the minimum necessary to allow the entry of the craniotome foot.

In general, a cranial bone window of about 2.0 to 2.5 cm in diameter can meet the requirements and be applied widely in most neurosurgical procedures [35, 38, 75]. In addition, the bone window used in keyhole surgery is elliptical in shape, not circular. Given a 2-cm bone flap, a variety of surgical instruments can be used simultaneously, such as brain retractors, suction tubes, bipolar coagulation instruments, and aneurysm clip appliers. If necessary, the neuroendoscopy could also be introduced into for observation via the keyhole craniotomy. Careful drilling of the inner bone edge could increase the angle for visualization and manipulation [35, 50, 53, 59, 75].

### Hospitalization and medical economics

A longer hospital stay is one of the main drawbacks of the standard craniotomy and clipping of intracranial aneurysms [49]. Keyhole surgery for intracranial aneurysms could reduce the length of the hospital stay [20]. Mori et al. reported that the mean duration of postoperative hospitalization for patients with unruptured middle cerebral artery aneurysms was 2.3 ± 3.4 days in the keyhole approach group, which was significantly less than those receiving standard craniotomy [49]. Radovanovic I reported another outcome in patients with unruptured aneurysms (keyhole group 1.55 ± 24 days vs conventional group 4.28 ± 0.71 days, *p* < 0.000,1) [61]. In addition, their research suggested that keyhole surgery resulted in shorter operative times both in unruptured (102.7 ± 4.35 vs 194.7 ± 10.26 min, *p* < 0.0001) and ruptured aneurysms (124.3 ± 827 vs 209 ± 13.84 min, *p* < 0.0001) [61]. As a result, the cost of the keyhole surgery had also been reduced (*p* < 0.0001) [61, 88].

### CSF drainage during or after a keyhole operation

Intraoperative strict hemostasis, small bone window, complete dural closure, and the limited soft tissue dissection make it unnecessary to place any drainage tube after keyhole surgeries [60]. However, for ruptured aneurysm with ventricular hematoma and intracerebral hemorrhage is another matter. For these patients, drainage tube can be used for bloody cerebrospinal fluid drainage [78, 83].

There may be a role for the drainage of CSF via lumbar drainage, before the procedure, as it allows in brain relaxation and helps in post-operative ICP reduction [23, 35, 38, 64].

## Summary conclusions

Keyhole microsurgery can be applied safely in experienced hands to a variety of different pathologies with acceptable results, which are similar to standard craniotomies. All these variants of keyhole approaches require careful preoperative planning, adequate patient selection, and detailed anatomical knowledge. This consensus is an attempt to guide step by step residents and young neurosurgeons to perform keyhole approaches while treating intracranial lesions.
